# Effect of Nitrogen Application on the Sensitivity of Desert Shrub Community Productivity to Precipitation in Central Asia

**DOI:** 10.3389/fpls.2022.916706

**Published:** 2022-07-18

**Authors:** Yong-Xin Zang, Wen-Xuan Xu, Ke Wu, Wei-Kang Yang

**Affiliations:** ^1^State Key Laboratory of Desert and Oasis Ecology, Xinjiang Institute of Ecology and Geography, Chinese Academy of Sciences, Urumqi, China; ^2^The Specimen Museum of Xinjiang Institute of Ecology and Geography, Chinese Academy of Sciences, Urumqi, China; ^3^Mori Wildlife Monitoring and Experimentation Station, Xinjiang Institute of Ecology and Geography, Chinese Academy of Sciences, Mori, China

**Keywords:** aboveground net primary productivity, nitrogen application, precipitation variation, desert ecosystem, sensitivity

## Abstract

Precipitation variability and nitrogen (N) deposition caused by anthropogenic activities could profoundly impact ecosystem productivity and carbon cycling. In desert ecosystems, vegetation is sensitive to changes in precipitation and N deposition. However, the impacts of large changes in precipitation, especially with a concurrent increase in N content, on plant community remain unclear. In this study, we carried out experiments to monitor the impacts of five precipitation levels and two N levels on the plant community function and composition from the Junggar desert in Central Asia during the period 2018–2019. Our results showed that: (1) Aboveground net primary production (ANPP) significantly increased with increasing precipitation, it followed a positive linear model under normal precipitation range, and nonlinear mode under extreme precipitation events; (2) N application led to an increase in ANPP, but did not significantly improve the sensitivity of ANPP to precipitation change; (3) Changes in N content and precipitation, and their impacts on ANPP were mainly driven by plant density. These results provide a theoretical basis for predict the future dynamics of terrestrial vegetation more accurately under climate change and increasing nitrogen deposition.

## Introduction

In arid and semi-arid areas, water shortage and nitrogen (N) deficit are often considered to be the two key factors limiting plant growth and community formation (Noy-Meir, 1973; Avolio et al., 2014). Human activities have intensified the terrestrial hydrological cycle (IPCC, 2018), leading to an increase in the frequency of extreme drought and extreme precipitation events (Swain et al., 2018). Moreover, they have also contributed to a 3–5 fold increase in active N over the past century (Gruber and Galloway, 2008; Boutin et al., 2017). Thus, such changes are bound to have profound effects on productivity and other ecosystem processes.

Aboveground net primary productivity (ANPP) is a functional indicator closely related to energy flux and carbon cycle (Melillo et al., 1993; Haberl et al., 2014). Therefore, the relationship between ANPP and precipitation has always been a long-term concern (Sala et al., 2012; Felton et al., 2019). Most research studies have shown that there is a positive asymmetry between ANPP and precipitation at the same location, indicating that the positive impacts of wet years on ANPP tend to be much greater than the negative impacts of dry years (Knapp and Smith, 2001; AhlstrÖm et al., 2015; Zhang et al., 2020). However, some studies also showed that there is a negative asymmetry (Luo et al., 2008; Wu et al., 2018) or a positive linear relationship (Wilcox et al., 2016; Felton et al., 2019) between ANPP and precipitation. In addition, these models do not fully consider extreme drought and extreme precipitation events; therefore, Knapp et al. (2017b) proposed a “non-linear double asymmetric” model, which has wider applications and considers both positive asymmetry under nominal precipitation conditions and negative asymmetry under extreme precipitation events.

As noted above, the relationship between ANPP and precipitation is inconsistent, and the differences in vegetation community composition, the sensitivity of species to precipitation change, and biogeochemical factors in different areas under study are the major reasons for the inconsistent relationship between ANPP and precipitation (Knapp et al., 2017b; Deng et al., 2021). Previous studies on the relationship between ANPP and precipitation focus primarily on temperate steppe ecosystems (Knapp et al., 2017a). As an important part of ecosystem system, desert ecosystems are areas that need urgent attention. However, the changing patterns of ANPP in desert ecosystems having a broad range of precipitation are still unclear (Knapp et al., 2017a; Song et al., 2019; de Boeck et al., 2020).

Besides precipitation, N has long been considered one of the major factors affecting plant growth, according to Liebig's law of the minimum (Thomas, 1929). Several studies have shown that N deposition could increase ANPP (Xu et al., 2016b; Liu et al., 2019), and the response of ANPP to N application is regulated by environmental factors, especially precipitation (Harpole et al., 2011). Precipitation could affect the mineralization rate and uptake of N; thus, regulating N turnover and its effect on plant community structure and function (Gebauer and Ehleringer, 2000). In turn, N application could also affect the sensitivity of plant community structure and function to precipitation change (Meng et al., 2021).

The synergistic interactions between multiple limiting resources have been widely studied (Harpole et al., 2011; Marleau et al., 2015). Those studies indicated that soil moisture and N content are the major factors limiting vegetation growth in most ecosystems. Therefore, N application generally increased the sensitivity of ANPP to increased precipitation (de klein et al., 2015; Ma et al., 2020). When compared to the vegetation community sensitivity to increased precipitation after N application, the sensitivity to reduced precipitation is still uncertain. N application could reduce the drought tolerance of plants by decreasing root shoot ratio and increasing stomatal conductance (Gessler et al., 2017), and improve the sensitivity of vegetation community to drought (Xu et al., 2014). However, certain mechanisms suggest a reduction in the sensitivity of ANPP to drought conditions after N application. For example, higher soil available N could increase the consumption of N-based compounds by plants for osmotic regulation, thus alleviating the nutrient deficiency caused by drought (He and Dijkstra, 2014). In recent years, nitrogen deposition has increased from 1.3 to 2.1 g N m^−2^ y^−1^ in China (Liu et al., 2013). Therefore, elucidating whether nitrogen application increases the sensitivity of ANPP to precipitation change will predict the future dynamics of terrestrial vegetation more accurately.

The mechanism governing the impact of precipitation and N content on ANPP is not yet well-understood (Weltzin et al., 2003; Ogle and Reynolds, 2004; Luo et al., 2021; Guo et al., 2022). Generally, the changes in precipitation determine the changes in ANPP by triggering physiological responses of individual plants and based on community attributes (plant density, height, species richness, etc.) (Smith et al., 2009). When there are changes in the precipitation pattern, the plant features initially affected are the leaf water potential and degree of stomatal opening and closing in plant leaves, thus affecting the photosynthetic capacity of plant leaves. Further, they could lead to a change in the biomass of individual plants by altering the plant height and tillering number (Weltzin et al., 2003; Yahdjian and Sala, 2006). However, this change is often limited. The demographic mechanisms suggest that the plant density increases to overcome meristem constraints and increase ANPP with the increase in precipitation, especially in arid and semi-arid regions (Hu et al., 2018; Felton et al., 2020). The diversity hypothesis suggests that greater species diversity leads to more functional strategies and complementary resource utilization to increase ANPP (Diaz and Cabido, 2001). However, most studies have shown that N deposition has increased ANPP but decreased species diversity (Siddique et al., 2010; Isbell et al., 2013b; Soons et al., 2017). Further studies are necessary to understand the effect of species diversity on productivity in resource-limited arid and semi-arid areas.

The Junggar Desert in Northwest China forms an important part of the temperate desert biome in Central Asia and has not been studied as much as deserts in other regions (Song et al., 2019). Similar to many drylands, the Junggar Desert is expected to experience an increased frequency of extreme drought and extreme precipitation events (Ma et al., 2015; Huang et al., 2017). Several studies have shown that water and N are the two major factors affecting vegetation productivity in the drylands (Yahdjian et al., 2011; Dijkstra et al., 2018). However, the effects of precipitation and N deposition and their impacts on vegetation productivity in arid ecosystems are not well-understood. In this context, the present study aims to analyze the sensitivity of plant community structure and functions to large changes in precipitation and to assess the influence of N application on ecosystem sensitivity. While conducting the study, we hypothesized that: (1) the relationship between ANPP of shrub communities in the Southeastern Junggar Desert and precipitation in the growing season conforms to the non-linear double asymmetric model, (2) N application increased the sensitivity of ANPP to precipitation change, and (3) ANPP was primarily driven by the plant density.

## Materials and Methods

### Study Site

The study was conducted in the Mori Wildlife Ecological Monitoring and Experimentation Station, Xinjiang Institute of Ecology and Geography, Chinese Academy of Sciences, located on the southeastern edge of the Junggar Basin, Northwest China (43°59′ N, 90°48′ E, 1,000 m AMSL). The climate of the study area is arid and cold with the monthly average temperature varying from-15°C in January to 20°C in July and annual precipitation of 150 mm. Winds are strong in spring and summer, and gales above force 7 are frequent and occur for over 80 days annually. The soil in the region is mainly clayey and sandy (Xu et al., 2016a). The vegetation is co-dominated by shrub species such as *Anabasis salsa* and *Seriphidium borotalensis* with the plant height ranging from 10 to 15 cm. The most common herbaceous plants found in the region are *Ceratocarpus arenarius, Salsola paulsenii, Halogeton glomeratus, and strigosella (Malcolmia) Africana* (Yang et al., 2008).

### Experimental Design

The experiments were conducted according to the split-plot design in randomized complete blocks. From early May 2016, the main plots were subjected to the precipitation treatment, while the subplots were treated with N. The precipitation treatment had five levels: one level as the precipitation control, i.e., treatment with the ambient amount of precipitation while the precipitation was reduced and increased by 60 and 30% relative to the ambient precipitation in the remaining four levels. The five levels are marked here as Control, −60, −30, +30, and +60%. The N treatment had two levels: without N application as the N control (N0) and with N application (N10) at the rate of 10 g N m^−2^ yr^−1^. This value is the critical load point to influence plant community structure and function (Bai et al., 2010). The main plots (8 × 3 m) subjected to the five-level precipitation treatment were randomly allocated to each block with five replicates, and the subplots (half of the main plot) subjected to the two-level N treatment were randomly placed in each main plot with a 3 m buffer zone separating contiguous main plots. During each early growing season (1–15 April), N was applied using ammonium nitrate (NH_4_NO_3_) dissolved in 33.75 L water (equal to 0.15 mm rainfall), and applied to each plot using a sprayer to distribute the fertilizer evenly. An equivalent amount of water was applied to each N control plot.

For drought plots, passive rainfall shelters were constructed to create experimental drought conditions based on the design given by Yahdjian and Sala (2002). We chose the same design as used in that study due to its effectiveness and because it exerted only minimal impacts on the energy balance and microclimatic conditions (Hoover et al., 2015). The shelters constructed were 0.6 m high at the short end and 1.6 m high at the long end. These rainout shelters were composed of 3 × 3 m steel and transparent polycarbonate plastic strips. An adequate buffer of 50 cm was set at the shelter edges to prevent the edge effects. The collected rainwater after each rainfall event was added manually to subplots which are to be treated with increased precipitation. The steel sheets were installed at the edges of each plot at a depth of 30 cm to prevent the horizontal exchange of soil water and nutrients between plots and isolated areas. The transparent polycarbonate plastic strips for the shelters will be removed in October and reinstalled in March of the following year.

### Precipitation Data

Long-term annual precipitation data were obtained from Drought-Net (http://www.drought-net.org/). During the growing season, the precipitation at the experimental site was obtained from an automatic meteorological observation station located about 50 m away from the experimental site. A lognormal function was used to calculate the estimated probability density function of precipitation.

### Vegetation Survey and Plant Aboveground Biomass

From April to September in the years 2018 and 2019, we recorded the number and height of plants were recorded twice a month, and calculated plant density and height using data from early August. The density of each plant species was calculated by dividing the number of plants by the plot area. The average plant height of the three densest species was considered the height of the community. Species richness was annually recorded as the total number of constituent plant species within the 1 m^2^ quadrat. Community cover was conducted using the method developed by Yang et al. (2011).

Aboveground biomass was measured during the peak biomass period in 2018 and 2019 (i.e., the first 2 weeks of August) by harvesting all the aboveground plant material in a 1 × 1 m quadrat in each plot. After harvesting, the shrubs and herbs were separated. All the plant samples were oven-dried at 75°C until the weights remained constant. We measured sensitivity as the change in ANPP per unit change in rainfall (Smith et al., 2017) and the sensitivity of shrubs and herbs were calculated as the mean of sensitivity from different precipitation treatments.

### Statistical Analyses

A generalized linear mixed model (GLMM) was used to evaluate the individual and interactive effects of precipitation change, N application, and year on community attributes (plant density, height, community cover, and species richness), ANPP and the sensitivity of ANPP. During the analyses, the precipitation change, N application, year, and their interactive effects were considered fixed factors, while plots were considered random effects. The model fitting was carried out by selecting the standard link function corresponding to the variables. Plant cover, density, height, ANPP and the sensitivity of ANPP data were found to be normally distributed. The species richness data showed binomial distribution and Poisson distribution. The standard link functions for these distributions are identity function, logit function, and logarithmic function, respectively (Zou et al., 2013). One-way ANOVA was used to determine the effect of different plant life forms on ANPP sensitivity.

The least significant difference (LSD; *P* < 0.05) test was used to compare the differences in the mean values when the GLMM and ANOVA results were significant. Regression analysis was used to evaluate the response of ANPP with precipitation change for N0 and N10. The final regression models were selected according to Akaike information criterion (AIC) and coefficient of determination (*R*^2^). All the data analyses were performed using SPSS 25.0 (SPSS Inc., Chicago, IL, USA), and the graphs were prepared using the software Origin 9.0 (OriginLab Corp., Northampton, MA, USA).

Finally, the structural equation modeling (SEM) technique was adopted to estimate the direct and indirect effects of precipitation change and N application and their integrated effects on ANPP using the Amos 21.0 software (Smallwaters Corporation, Chicago, IL, USA) based on the hypothesized causal relationships and the results from the previous studies (Supplementary Figure 1). The fitness of the SEM model was evaluated by the non-significant χ^2^ test (*P* > 0.05), low Akaike value (AIC), high goodness of fit index (GFI) (>0.90), and low root mean square error of approximation (RMSEA) (<0.05) (Hooper et al., 2008).

## Results

### Changes in Precipitation

**Figure 1:**
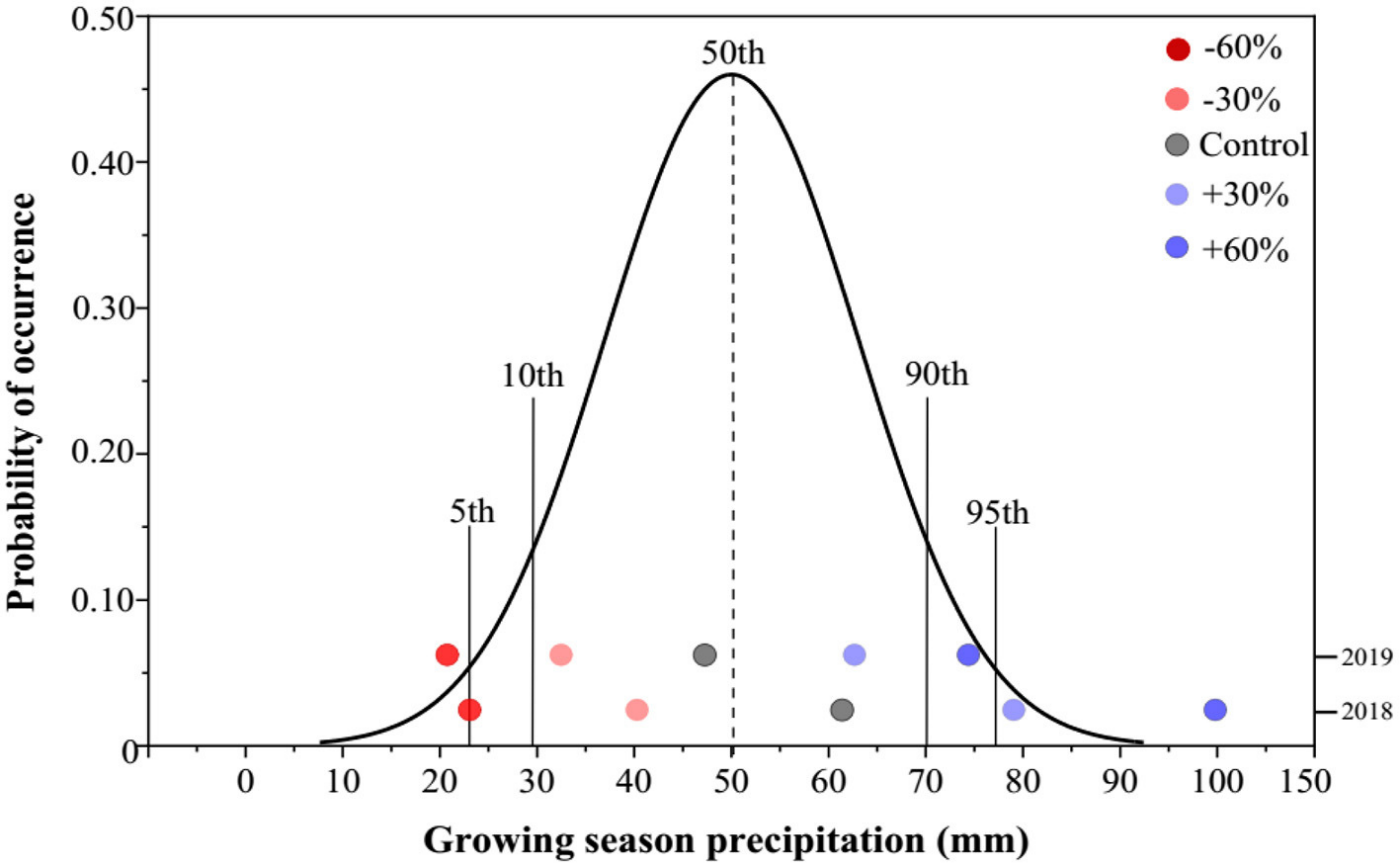
Probability distribution of annual precipitation in the study area during the plant growing season over the past 100 years. The precipitation treatments include five levels, namely, −60%, −30%, Control, +30%, and +60% precipitation relative to the ambient level.

Based on the meteorological data from the study area, the mean growing-season (end of March to September) precipitation (GSP) was 50.1 mm, whereas the GSP in 2018 and 2019 was 62.5 and 47.7 mm, respectively (Figure 1). The mean GSP values for −60 and +60% precipitation treatments in our study were 25.0 and 100.0 mm, respectively, in 2018. The former GSP value was marginally near the 10th percentile, while the latter exceeded the 95th percentile of the historical GSP distribution. In 2019, the GSP of −60% precipitation treatment was reduced to 19.1 mm, below the 5th percentile, whereas the GSP of +60% precipitation treatment increased to 76.^*^3 mm, exceeding the 90th percentile (Figure 1).

### Changes in Community Attributes

**Figure 2:**
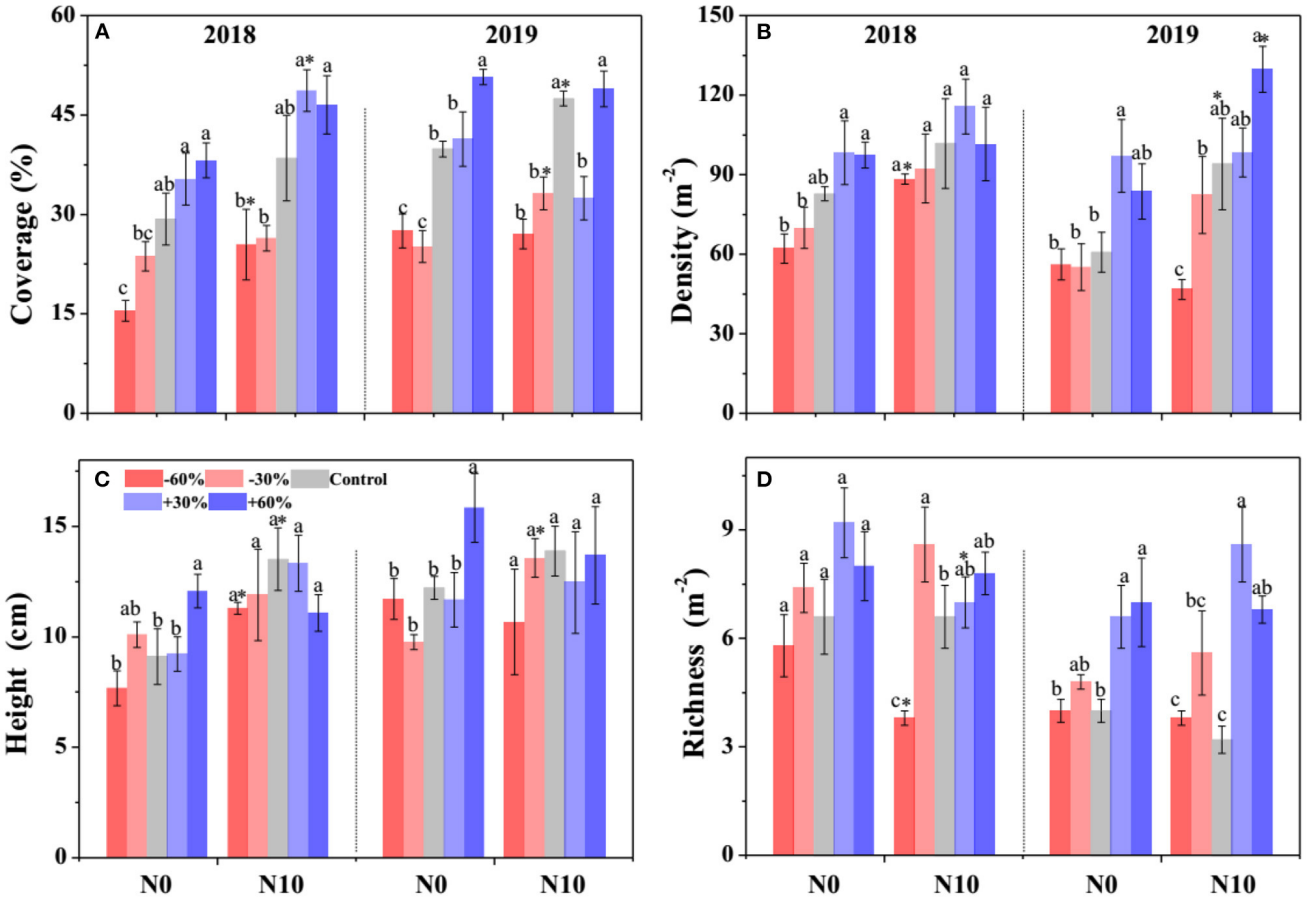
Effects of precipitation change and nitrogen application on community attributes **(A–D)** in 2018 and 2019. Bars are represented as mean ±1 SE. Different lowercase letters indicate significant differences (*P* < 0.05) between precipitation treatment groups. *Indicates significant differences (*P* < 0.05) between the N0 and N10 treatments. The precipitation treatments include five levels, namely, −60%, −30%, Control, +30%, and +60% precipitation relative to the ambient level. N0 and N10 represent the treatments without N addition (Control) and with 10 g N m^−2^ yr^−1^ application of nitrogen, respectively.

According to the GLMM, precipitation, N addition, and year led to significant changes in the community cover, plant density, height, and species richness (Table 1). Community cover, plant density, height, and species richness increased gradually with the increasing precipitation (Figure 2). Precisely, −60, −30, +30, and +60% precipitation treatments led to −38.7, −29.4, +4.7, and +20.3% change in community cover; −23.9, −11.6, +24.3, and +23.3% change in plant density; −14.5, −5.8, −3.7, and +10.7% change in height and −8.9, −34.3, +69.8, and +56.7.0% change in species richness relative to the control, respectively (Figure 2). N addition also led to a notable (*P* < 0.05) increase in the community cover (+15.7 %), plant density (+15.7 %), and height (+22.4 %); however, it caused a significant (*P* < 0.05) decline in the species richness (−4.4%) (Figure 2). In addition, the interactive effect of precipitation and N application led to significant changes in community plant height, density and species richness (Table 1). N addition promoted the increase of plant density and height induced by precipitation, but inhibited the increase of species richness (Figure 2).

**Table 1:** GLMM results for the effects of precipitation change (P), nitrogen application (N), year (Y), and their interactive effects on ANPP, the sensitivity of ANPP (Sensitivity) and community attributes.

**Effect**	**Cover**		**Density**	**Height**	**Richness**	**ANPP**	**Sensitivity**	
	**Df**	***F***	***F***	***F***	***F***	***F***	**df**	***F***
P	4	11.14^**^	36.89^**^	40.52^**^	47.74^**^	19.37^**^	3	1.8
N	1	4.49^*^	185.22^**^	173.07^**^	173.26^**^	78.56^**^	1	1.31
Y	1	10.94^**^	18.92^**^	24.99^**^	70.66^**^	0.16	1	0.05
P × N	4	0.56	2.76^*^	6.85^**^	3.01^*^	0.17	3	1.03
P × Y	4	3.43^*^	4.35^**^	1.94	7.16^**^	0.08	3	0.06
N × Y	1	7.86^**^	0.14	9.30^**^	6.05^*^	0.07	1	0.02
P × N × Y	4	2.76^*^	7.33^**^	3.13^*^	5.61^**^	0.12	3	0.09

* Indicates P < 0.05 and ^**^indicates P < 0.01.

### Relationship Between Precipitation and ANPP After N Application

**Figure 3:**
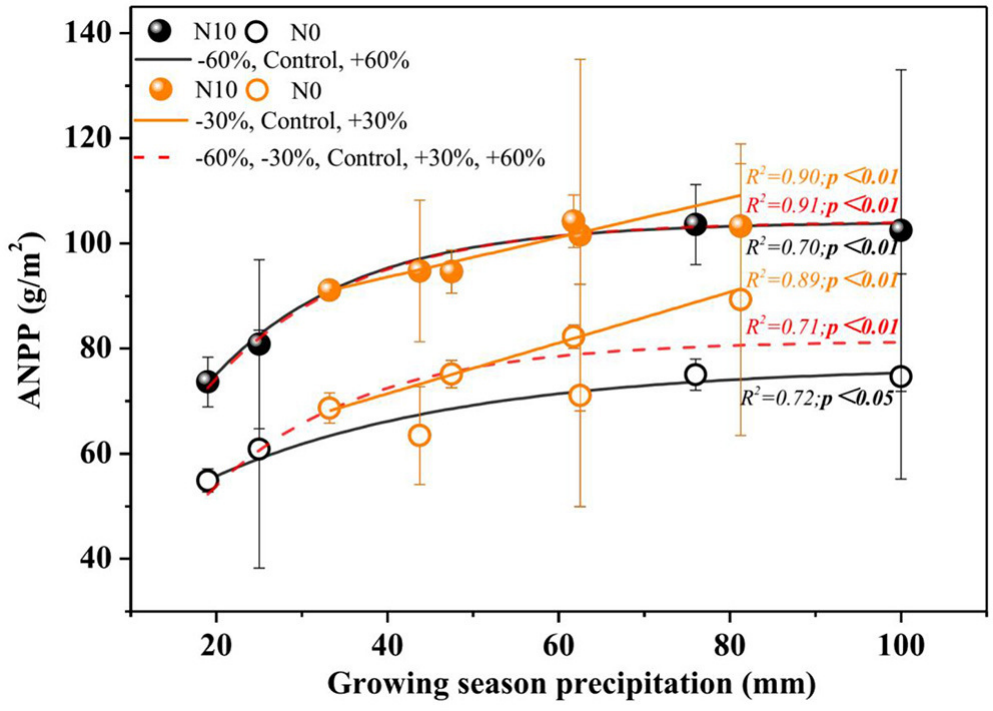
Relationship between ANPP and amount of precipitation under two nitrogen levels. *P-values* smaller than 0.05 are presented in bold. Points are represented as mean ±1 SE. The precipitation treatments include five levels, namely, −60%, −30%, Control, +30%, and +60% precipitation relative to the ambient level. N0 and N10 represent the treatments without N addition (Control) and with 10 g N m^−2^ yr^−1^ application of nitrogen, respectively. The fitting function is shown in Supplementary Table 1.

Precipitation and N application resulted in significant changes in ANPP (Table 1, Figure 3, *P* < 0.01). Precipitation treatments led to a significant change in ANPP (Figure 3). More specifically, −60, −30, +30, and +60% precipitation treatments led to −20.9, −7.4, +11.7, and +3.8% change in ANPP relative to the control, respectively (Figure 3). N addition also led to a notable increase (+25.0%) in the ANPP (Figure 3).

**Figure 4:**
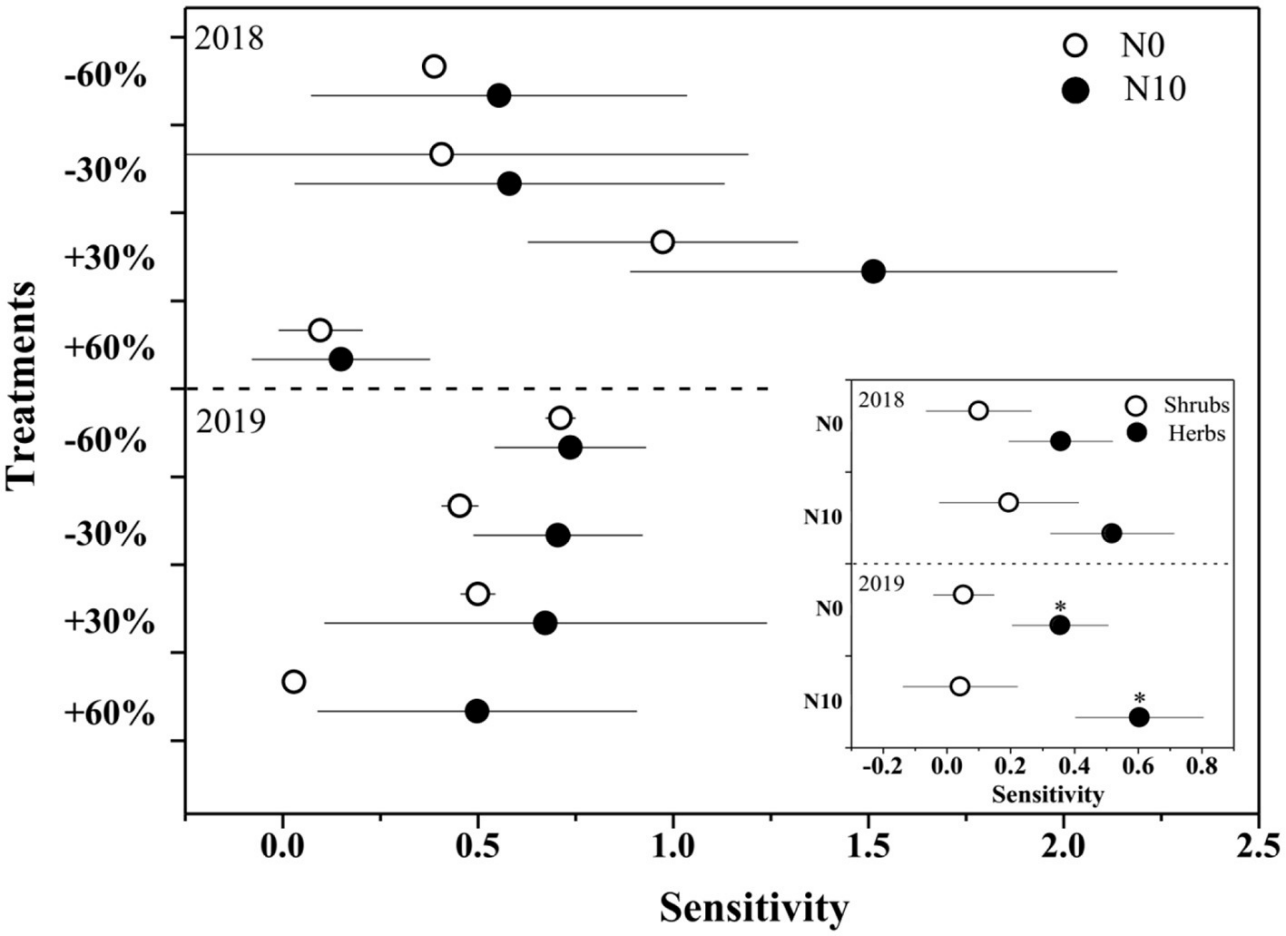
The sensitivity of ANPP and shrubs and herbs (inserted panels) to precipitation change and nitrogen application in 2018 and 2019. Points are represented as mean ±1 SE. The precipitation treatments include five levels, namely, −60%, −30%, Control, +30%, and +60% precipitation relative to the ambient level. N0 and N10 represent the treatments without N addition (Control) and with 10 g N m^−2^ yr^−1^ application of nitrogen.

In the normal precipitation range, ANPP increased gradually with increasing precipitation, and a linear model was fitted better than nonlinear model (Figure 3 and Supplementary Table 1). However, a nonlinear model was fitted better than linear model for the ANPP and precipitation relationship under extreme precipitation events and all precipitation gradients (Figure 3 and Supplementary Table 1). Although N application increased the sensitivity of ANPP to precipitation change, it was not significant (Figure 4 and Table 1). N application did not change the fitting relationship between ANPP and precipitation, that is, the fitting relationship between ANPP and precipitation is a linear model in the normal precipitation range, and nonlinear model in the extreme precipitation events and all precipitation gradients (Figure 3).

With precipitation application from 19.1 to 100.0 mm, the relative ANPP of shrubs declined from 77.0 to 51.1%, while the relative ANPP of herbaceous plants increased from 22.9 to 40.8% (Supplementary Figure 2). N application further increased the proportion of herbaceous plants (19.1 mm: from 22.9 to 31.7%; 100.0 mm: from 40.5 to 48.8%, Supplementary Figure 2). In addition, the sensitivity of ANPP of herbaceous plants to precipitation change was significantly (*F* = 96.2, *P* < 0.01) greater than that of shrubs (Figure 4). N addition increased the sensitivity of herbaceous and shrub ANPP to precipitation change, but neither of them was significant (Figure 4).

### Effects of Precipitation and N Application on ANPP

**Figure 5:**
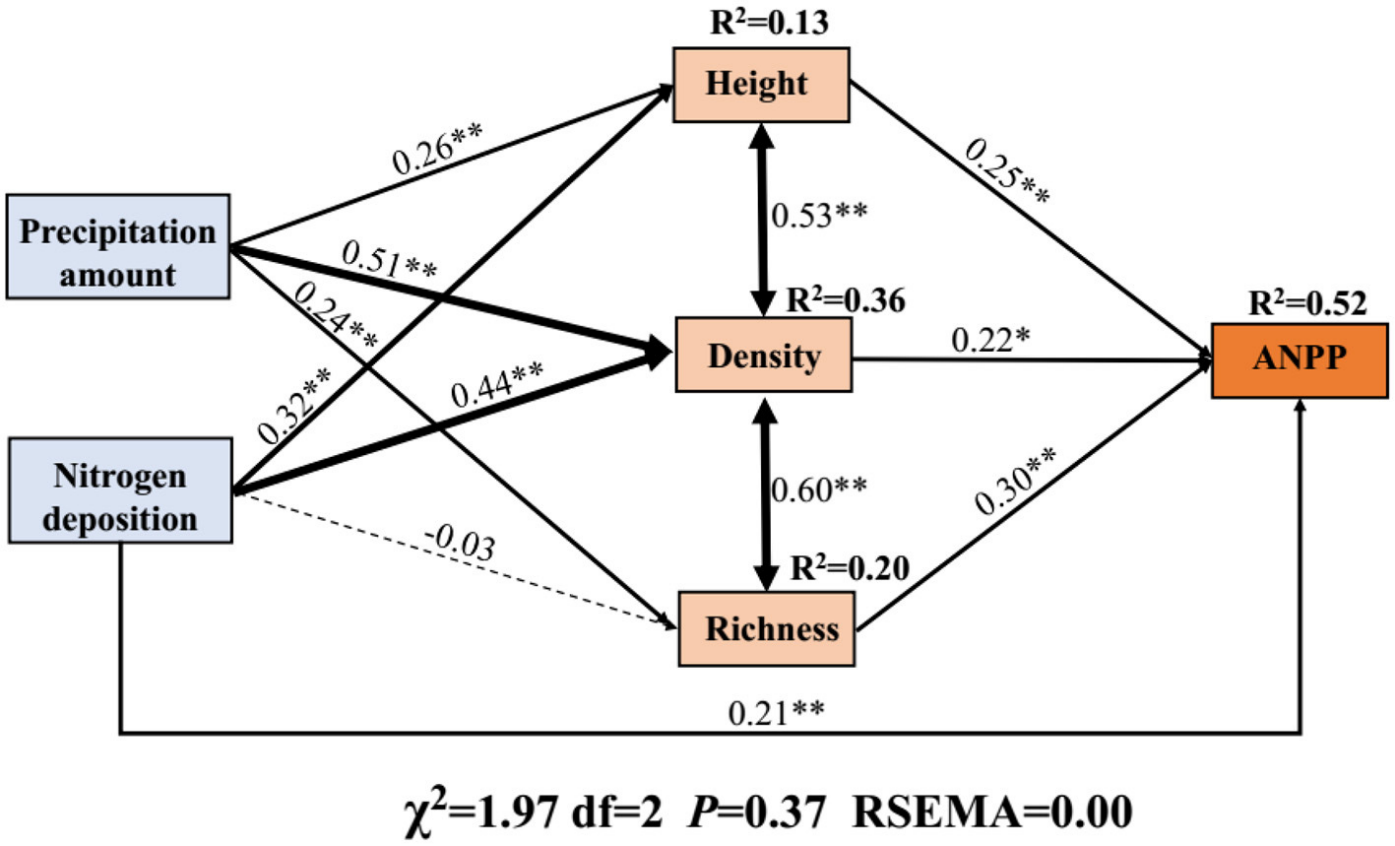
Structural equation model (SEM) representing the effects of precipitation change, nitrogen application, and their integrated impacts on ANPP. The SEM considered all plausible pathways through which plant traits influence ANPP. Solid lines represent the positive paths, and dashed lines indicate negative paths. Arrow width is proportional to the strength of the relationship. *Indicates *P* < 0.05 and **indicates *P* < 0.01. *R*^2^ represents the proportion of the variance for each dependent variable in the model.

The SEM was used to assess the integrated effects of precipitation, N application, and community attributes (plant density, height, and species richness) on ANPP. Overall, the precipitation and N application increased the ANPP mainly by increasing the plant density (Figure 5). Further, quantification of the indirect effects (product of path coefficients) implied that the major factor affecting ANPP was plant density (0.112), followed by species richness (0.072), and height (0.065), as shown in Figure 5. N application increased the ANPP also mainly by increasing the plant density (0.097) followed by height (0.080, Figure 5). In addition, N application had a negative effect on species richness (Figure 5).

## Discussion

### The Relationship Between ANPP of Shrub Communities and Precipitation in the Growing Season

In this study, the relationship between ANPP and precipitation is not completely consistent with our hypothesis. In the normal precipitation range, a linear model was fitted better than nonlinear models for the ANPP and precipitation relationship (Figure 3). Both recent studies conducted in the Central Great Plains of Colorado, USA and Damao County, Inner Mongolia, China, showed that linear relationship often best explains the ANPP responses to changes in precipitation (Felton et al., 2019; Ma et al., 2020), and they suggested that the rapid plant compositional shifts may be the reason for the linear relationship between ANPP and precipitation.

Our results are consistent with non-linear double asymmetric model proposed by Knapp et al. (2017b) only under extreme precipitation events, that is, the increase of ANPP of extreme precipitation is less than the decrease of extreme drought (Figure 3). In general, plant physiological processes, meristem tillering, density constraints and soil nutrients could well account for the non-linear double asymmetric model in both extreme drought and extreme precipitation (Yahdjian and Sala, 2006; Smith et al., 2009; Xu and Zhou, 2011; Felton et al., 2019).

The “hierarchical response framework” suggested by Smith et al. (2009) postulates that general drought conditions could trigger the physiological response of plants. However, drought conditions for a long time or extreme drought conditions might lead to plant physiological responses exceeding the threshold limit. Such extreme conditions result in a significant increase in community mortality, a decrease in the plant density (Niu et al., 2014; Felton and Smith, 2017). The results of our study showed that plant density was the most important driving factor of ANPP (Figure 5). Therefore, the decrease of plant density caused by extreme drought would inevitably increase the rate of ANPP decline (Figures 2B, 3).

In addition, according to Liebig's law of the minimum (Thomas, 1929), the factors limiting plant growth are often the ones present in small quantities relative to the plant demand. Therefore, when there are extreme precipitation events, soil nutrient distribution may become the primary factor limiting plant growth in general, especially in N-deficient desert ecosystems (Huxman et al., 2004; Eskelinen and Harrison, 2015). As a result, the rate of ANPP rise declines, and the relationship between ANPP and precipitation shows a negative asymmetry during extreme precipitation.

The experimental area under study is characterized by poor soil nutrient content (Wu et al., 2020). However, interestingly, N application did not change the relationship between ANPP and precipitation in the growing season, which still made a nonlinear model was fitted better than linear model for the ANPP and precipitation under N10 treatments (Figure 3).

In addition to soil nutrients, vegetation constraints may also play an important role in mediating ANPP-precipitation relationship in the drylands (Lauenroth and Sala, 1992; Sala et al., 2012; Felton et al., 2019). Vegetation constraints, such as leaf area, meristem, tiller density, and overall growth limitations responses to water availability is a key mechanism limiting ANPP, particularly with respect to responses to increases in precipitation (Yahdjian and Sala, 2006; Estiarte et al., 2016; Knapp et al., 2017b). Unfortunately, our analyses did not include these indicators (e.g., leaf area, meristem). Moreover, the community composition discussed in detail below may also be one of the important factors regulating the relationship between ANPP and precipitation.

### Interactive Effects of Precipitation Change and N Application on ANPP

In contrast to our hypothesis, although N application increased ANPP (Figure 3 and Table 1), it did not significantly improve the sensitivity of ANPP to precipitation change (Figure 4 and Table 1). Terrestrial ecosystems are generally limited by nitrogen, so increased nitrogen could improve community productivity (Hooper and Johnson, 1999; Vitousek et al., 2002; Xu et al., 2016b; Liu et al., 2019). Furthermore, several recent studies have demonstrated that N deposition could also improve the sensitivity of ANPP to precipitation reduction or increase (Bharath et al., 2020; Ma et al., 2020; Meng et al., 2021).

N application not only increases the nutrient availability in the soil but also decreases the drought tolerance of plants by reducing the root to shoot ratio, increasing the stomatal conductance and plant height, and impairing the symbiotic relationship between plants and mycorrhiza (Gessler et al., 2017). Xu et al. (2014) found that long-term N application resulted in a 33% increase in the grassland productivity response to drought conditions in Duolun Grassland, Inner Mongolia, China. The multiple resource co-limitation theory proposed by Harpole et al. (2011) states that soil N dynamics and the effect of N on plant communities largely depend on water availability (Delgado-Baquerizo et al., 2013; Xu et al., 2016b). Therefore, N application generally increased the sensitivity of ANPP to increased precipitation (de klein et al., 2015). While, in the present study, N application did not significantly improve the sensitivity of ANPP to precipitation change (Figure 4), which may be related to community composition.

The dominant species in our study area are shrubs *A. salsa* and *S. borotalensis*. In general, desert shrubs, as perennial woody plants, grow at a slow rate and have stronger resistance to stress and poor environment than herbaceous plants, therefore, they are relatively insensitive to environmental changes (Peters et al., 2012; Baze et al., 2013). Gonzalez-Paleo and Ravetta (2018) found that shrubs are less sensitive to high levels of nitrogen availability than annual herbs with acquisitive resource use strategies. Therefore, nitrogen-induced changes in herbs productivity tend to account for most of the total changes in ANPP in arid shrub communities (Hall et al., 2011; Peters et al., 2012). A recent meta-analysis (Xu, 2021) also found that the response of ANPP of woody plants (36.15%) to nitrogen addition was significantly lower than that of herbaceous plants (56.11%). In this study, our results also showed that herbaceous plants were significantly more sensitive to precipitation change and N application than shrubs (Figure 4). However, according to the mass ratio hypothesis (Grime, 1998), dominated species occupy the majority of the niche space and have disproportionate effects on ecological processes, so the change of community productivity mainly depends on the dominant species (Smith et al., 2020). Therefore, due to the large proportion of shrubs biomass (Supplementary Figure 2), N application did not significantly improve the sensitivity of community ANPP to precipitation change.

The evidence above suggests that shrub-herb composition, rather than soil nutrients, may be the main factors regulating the sensitivity of community productivity to precipitation change and ANPP-precipitation relationship in the arid shrub communities.

### Precipitation Change and N Application Drive ANPP Mainly Through Plant Density

Our results showed that plant density had the greatest effect on ANPP under precipitation change and N application (Figure 5). The results confirmed our hypothesis. It is well-known that ANPP is closely related to the plant density, height (Smith et al., 2009) and species richness (Hooper et al., 2005; Isbell et al., 2013a), and precipitation and N application change the values of these parameters, further driving changes in ANPP (Zang et al., 2021).

Plant height is a trait associated with plant growth ability, so community productivity usually increases with plant height (Moles et al., 2009; Luo et al., 2021). However, in our study area, winds are strong in the growing season, and gales above force 7 are frequent and occur for over 80 days annually, which leads to plants widespread dwarfing (Zhang et al., 2013; Xu et al., 2017). Therefore, the precipitation and nitrogen could only increase plant height limited. The seeds in deserts have flexible germination strategies due to high precipitation variability and unpredictability (Noy-Meir, 1973). They can quickly acclimatize to the external environment and make adjustments with respect to the germination time (Koller, 1972; Chen et al., 2020). With increased precipitation, higher germination of seeds was observed, led to an increase in species richness and plant density (Figures 2B,D and Table 1). Many biodiversity ecosystem functioning experiments have shown that community productivity increases with the species richness, and the species added are generally rare species (Duncan and Young, 2000; Tilman et al., 2001; Hooper et al., 2012). Although rare species can contribute significantly to total community diversity, their contribution to total primary production is relatively low (Smith et al., 2020).

Two major factors may be responsible for plant density had the greatest effect on ANPP under precipitation change and N application. On the one hand, as mentioned above, when a long time drought conditions or extreme drought occurs, the threshold of plant physiological response is broken, and the mortality rate of community increases and the plant density decreases, leading to the decline of ANPP (Smith et al., 2009; Niu et al., 2014; Felton and Smith, 2017). On the other hand, when the availability of water and nitrogen resources increases significantly, the demographic mechanisms suggest that communities increase ANPP mainly by increasing plant density to overcome meristem limitations, especially in desert ecosystems with limited meristem and tillering potential (Hu et al., 2018; Felton et al., 2020).

## Conclusions

In the present study, field experiments were conducted considering water and N as treatment factors. The study attempts to assess the potential mechanisms by which large precipitation change and N application elicit a notable ecological response. In addition, the interactive effects of precipitation and N application were also evaluated. The study revealed that the relationships between ANPP and precipitation conform to a linear model under normal precipitation range, and nonlinear mode under extreme precipitation events. N application increased ANPP, but it did not significantly improve the sensitivity of ANPP to precipitation change. Moreover, the individual effects of N application and precipitation and their integrated effects on ANPP were mainly driven by plant density, rather than by height or species richness. The ecosystem sensitivity to future changes in precipitation may be regulated by N application. Thus, it can be concluded that multiple constraints should be considered while assessing the sensitivity of terrestrial ecosystems to climate change.

## Data Availability Statement

The raw data supporting the conclusions of this article will be made available by the authors, without undue reservation.

## Author Contributions

W-KY and W-XX designed the experiment. KW and Y-XZ performed the field and laboratory work. Y-XZ analyzed the data. Y-XZ and W-XX wrote the manuscript. W-KY provided valuable comments and suggestions on draft. All authors discussed the results and commented on the manuscript.

## Funding

This work was supported by the National Natural Science Foundation of China (Nos. U2003214 and 31872254), Biodiversity Monitoring and Research Network Project of the Chinese Academy of Sciences (Sino-BON), the Strategic Priority Research Program of the Chinese Academy of Sciences (No. XDA2005020402).

## Conflict of Interest

The authors declare that the research was conducted in the absence of any commercial or financial relationships that could be construed as a potential conflict of interest.

## Publisher's Note

All claims expressed in this article are solely those of the authors and do not necessarily represent those of their affiliated organizations, or those of the publisher, the editors and the reviewers. Any product that may be evaluated in this article, or claim that may be made by its manufacturer, is not guaranteed or endorsed by the publisher.
