# Supplementary material for: Effect of Nitrogen Application on the Sensitivity of Desert Shrub Community Productivity to Precipitation in Central Asia
Source: Front Plant Sci. 2022 Jul 18;13:916706. doi: 10.3389/fpls.2022.916706 (PMC9340062; doi:10.3389/fpls.2022.916706)

**TABLE S1**. Fitting function of growing season precipitation and ANPP under N0 and N10 treatments. The precipitation treatments include five levels, namely, -60%, -30%, Control, +30%, and +60% precipitation relative to the ambient level. N0 and N10 represent the treatments without N addition (Control) and with 10 g N m^–2^ yr^–1^ application of nitrogen, respectively.

| Treatments | | | Fitting function | *R^2^* | AIC |
| --- | --- | --- | --- | --- | --- |
| N0 | -30%, +30% | | y=621654.7-23088.6.1exp (-(x-1.0)/1.1) | 0.16 | 7.24 |
|  |  |  | y=52.09+0.48x | 0.89 | -4.52 |
|  | -60%, +60% | | y=76.7-20.7exp (-(x-20.4)/29.2) | 0.72 | -10.12 |
|  |  |  | y=52.2+0.38x | 0.46 | 7.41 |
|  | -30%, -60%, +30%, +60% | | y=81.5-24.5exp (-(x-24.5)/17.9) | 0.71 | 6.55 |
|  |  |  | y=50.6+0.44x | 0.29 | 13.82 |
| N10 | -30%, +30% | | y=104.5-13.2exp (-(x-33.9)/9.7) | 0.03 | -9.34 |
|  |  |  | y=78.5+0.37x | 0.90 | -10.12 |
|  | -60%, +60% | | y=104.0-29.9exp (-(x-19.4)/16.7) | 0.90 | -22.71 |
|  |  |  | y=65.5+0.54x | 0.89 | -10.12 |
|  | -30%, -60%, +30%, +60% | | y=104.2-30.2exp (-(x-19.8)/16.9) | 0.91 | -36.81 |
|  |  |  | y=75.1+0.44x | 0.70 | -3.61 |

**Figure S1**. A priori structural equation model (SEM) relating proximate, intermediate and distal predictors to the final responses of community productivity to precipitation changes and nitrogen addition. Boxes represent measured variables and arrows represent hypothesized causal relationships among variables. The citations for all hypothetical paths are provided.


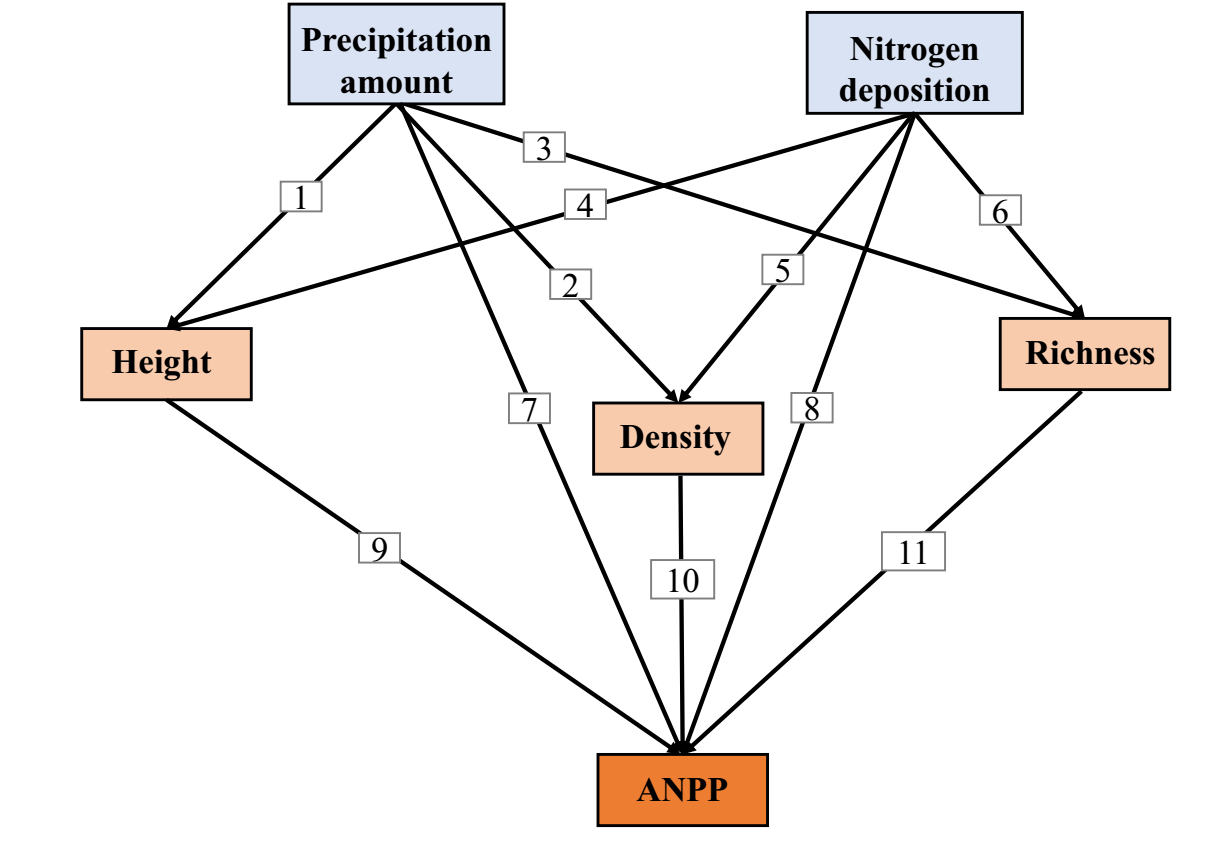


| **Pathway number** | **Direction** | **Supporting references** |
| --- | --- | --- |
| 1.Precipitation amount →Height | + | Zang et al., 2020; Guo et al., 2022 |
| 2.Precipitation amount →Density | + | Hoover et al., 2015; Zang et al., 2021 |
| 3.Precipitation amount →Richness | + | Palmquist et al., 2014; Muraina et al., 2021 |
| 4.Precipitation amount →ANPP | + | Hoover et al., 2014; Meng et al., 2021 |
| 5.Nitrogen deposition →Height | + | Wu et al., 2008; Ma et al, 2019 |
| 6.Nitrogen deposition →Density | + | Poorter et al., 2012; Demalach, 2018 |
| 7.Nitrogen deposition →Richness | - | Stevens et al., 2004; Zhang et al., 2019 |
| 8.Nitrogen deposition →ANPP | + | Ma et al, 2019; Guo et al., 2022 |
| 9.Height →ANPP | + | Fan et al, 2014; Zang et al., 2021 |
| 10.Density →ANPP | + | Hoover et al., 2015; Zang et al., 2021 |
| 11.Richeness →ANPP | + | Palmquist et al., 2014; Muraina et al., 2021 |

Demalach, N. (2018). Toward a mechanistic understanding of the effects of nitrogen and phosphorus additions on grassland diversity. Perspect Plant Ecol. 32: 65-72. doi: 10.1016/j.ppees.2018.04.003

Fan, L.L., Tang, L.S., Wu, L.F. (2014). The limited role of snow water in the growth and development of ephemeral plants in a cold desert. J Veg Sci. 25, 681-690. doi: 10.1111/jvs.12121

Guo, X., Zuo, X., Yue, P., Li, X., Hu, Y. (2022). Direct and indirect effects of precipitation change and nutrients addition on desert steppe productivity in Inner Mongolia, Northern China. Plant Soil. 1-14. doi: 10.21203/rs.3.rs-244027/v1

Hoover, D.L., Knapp, A.K., Smith, M.D. (2014). Resistance and resilience of a grassland ecosystem to climate extremes. Ecology. 95, 2646–2656. doi: 10.1890/13-2186.1.

Hoover, D. L., Duniway, M. C., Belnap, J. (2015). Pulse-drought atoppress-drought: unexpected plant responses and implications for dryland ecosystems. Oecologia. 179, 1211–1221. doi: 10.1007/s00442-015-3414-3

Ma, Q.H., Liu, X.D., Li, Y.B., Li, L., Yu, H.Y., Qi, M. (2020). Nitrogen deposition magnifies the sensitivity of desert steppe plant communities to large changes in precipitation. J Ecol. 108(2), 598–610. doi: 10.1111/1365-2745.13264

Meng, B., Li, J., Zhong, S., Maurer, G., Yao, Y., Yang, X., Collins, S., Sun, W. (2021). Nitrogen addition amplifies the nonlinear drought response of grassland productivity to extended growing-season droughts. Ecology. 102(11), e03483. doi: 10.1002/ecy.3483

Muraina, T.O., Xu, C., Yu, Q., Yang, Y.D., Jing, M.H., Jia, X.T. (2020). Species asynchrony stabilises productivity under extreme drought across Northern China grasslands. J Ecol. 109, 1665-1675. doi: 10.1111/1365-2745.13587

Palmquist, K.A., Peet, R.K., Weakley, A.S. (2014). Changes in plant species richness following reduced fire frequency and drought in one of the most species-rich savannas in North America. J Veg Sci. 25: 1426-1437. doi: 10.1111/jvs.12186

Poorter, H., Niklas, K.J., Reich, P.B. (2012). Biomass allocation to leaves, stems and roots: meta‐analyses of interspecific variation and environmental control. New Phytol. 193(1): 30-50. doi: 10.1111/j.1469-8137.2011.03952.x

Stevens, C.J., Dise, N.B., Mountford J.O., Gowing, D.J. (2004). Impact of nitrogen deposition on the species richness of grasslands. Science. 303:1876–1879. doi: 10.1126/science.1094678

Wu, F., Bao, W., Li, F. (2008). Effects of drought stress and N supply on the growth, biomass partitioning and water-use efficiency of *Sophora davidii* seedlings. Environ Exp Bot. 63(1): 248-255. doi: 10.1016/j.envexpbot.2007.11.002

Zang, Y.X., Min, X.J., de Dios, V.R., Ma, J.Y., Sun, W. (2020). Extreme drought affects the productivity, but not the composition, of a desert plant community in Central Asia differentially across microtopographies. Sci Total Environ, 717, 137251. doi: 10.1016/j.scitotenv.2020.137251

Zang, Y.X., Ma, J.Y., Zhou, X.B., Tao, Y., Yin, B.F., Zhang, Y.M. (2021). Extreme precipitation increases the productivity of a desert ephemeral plant community in Central Asia, but there is no slope position effect. J Veg Sci. 32: e13077. doi: 10.1111/jvs.13077

Zhang, Y., Feng, J., Loreau, M., He, N., Han, X., Jiang, L. (2019). Nitrogen addition does not reduce the role of spatial asynchrony in stabilising grassland communities. Ecol Lett. 22:563–571. doi: 10.1111/ele.13212

**Figure S2**. The ratio to the ANPP of the shrubs and herbs in 2018 and 2019. The precipitation treatments include five levels, namely, -60%, -30%, Control, +30%, and +60% precipitation relative to the ambient level. N0 and N10 represent the treatments without N addition (Control) and with 10 g N m^–2^ yr^–1^ application of nitrogen.


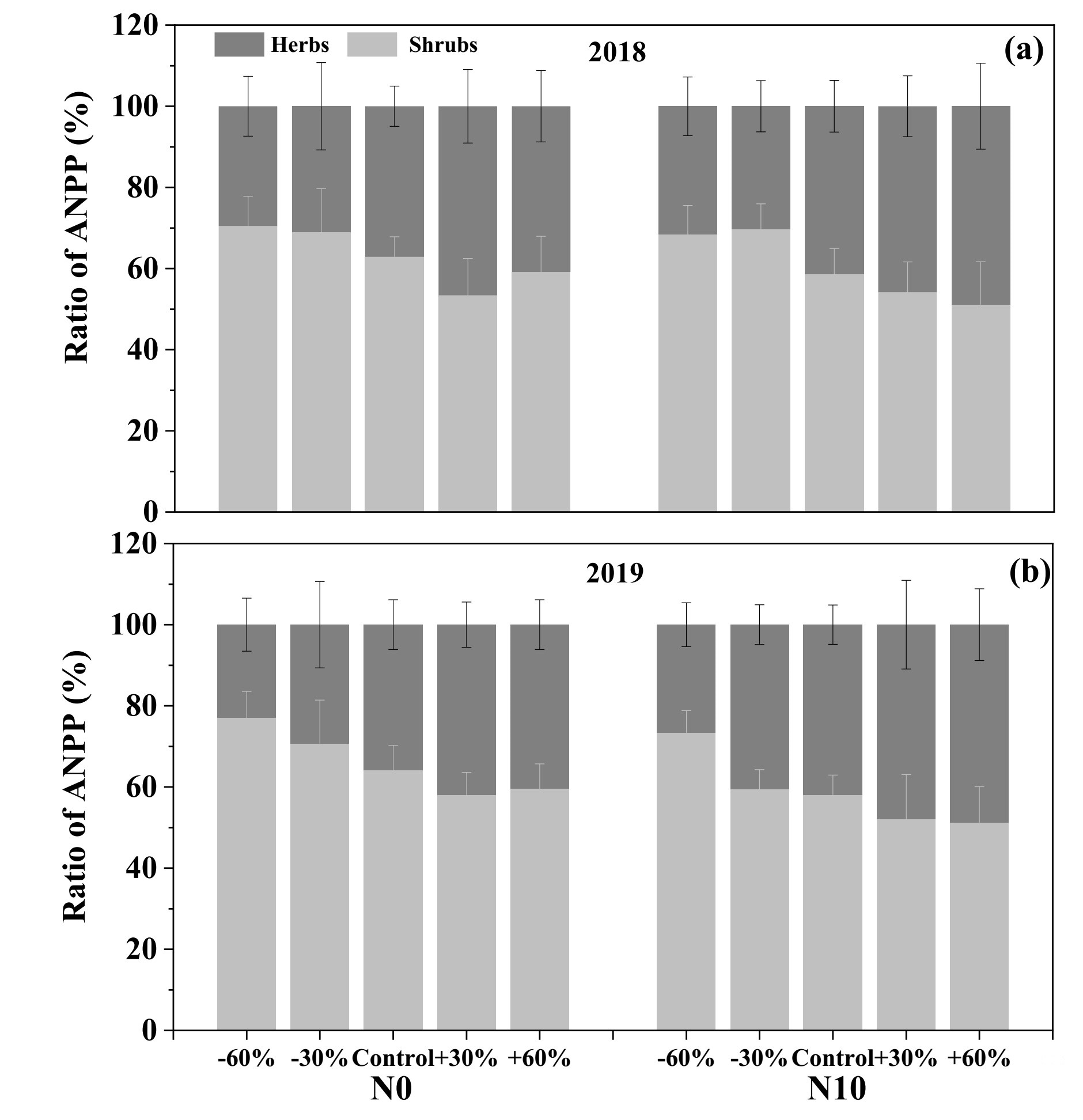

Supplement: Supplementary file 1 [file Data_Sheet_1.docx]
